# Genetic and genomic basis of antibody response to porcine reproductive and respiratory syndrome (PRRS) in gilts and sows

**DOI:** 10.1186/s12711-016-0230-0

**Published:** 2016-07-14

**Authors:** Nick V. L. Serão, Robert A. Kemp, Benny E. Mote, Philip Willson, John C. S. Harding, Stephen C. Bishop, Graham S. Plastow, Jack C. M. Dekkers

**Affiliations:** Department of Animal Science, Iowa State University, Ames, IA 50011 USA; Department of Animal Science, North Carolina State University, Raleigh, NC 27695 USA; Genesus Inc., Oakville, MB R0H 0Y0 Canada; Department of Animal Science, University of Nebraska-Lincoln, Lincoln, NE 68583 USA; Canadian Centre for Health and Safety in Agriculture, University of Saskatchewan, Saskatoon, SK S7N 2Z4 Canada; Department of Large Animal Clinical Sciences, University of Saskatchewan, Saskatoon, SK S7N 5B4 Canada; The Roslin Institute and Royal (Dick) School of Veterinary Studies, University of Edinburgh, Easter Bush, Midlothian, EH25 9RG UK; Department of Agricultural, Food and Nutritional Science, University of Alberta, Edmonton, AB T6G 2R3 Canada

**Keywords:** Disease resistance, Disease resilience, Genomic prediction, Genomic selection, PRRSV, Vaccination

## Abstract

**Background:**

Our recent research showed that antibody response to porcine reproductive and respiratory syndrome (PRRS), measured as sample-to-positive (S/P) ratio, is highly heritable and has a high genetic correlation with reproductive performance during a PRRS outbreak. Two major quantitative trait loci (QTL) on *Sus scrofa* chromosome 7 (SSC7; QTL_MHC_ and QTL_130_) accounted for ~40 % of the genetic variance for S/P. Objectives of this study were to estimate genetic parameters for PRRS S/P in gilts during acclimation, identify regions associated with S/P, and evaluate the accuracy of genomic prediction of S/P across populations with different prevalences of PRRS and using different single nucleotide polymorphism (SNP) sets.

**Methods:**

Phenotypes and high-density SNP genotypes of female pigs from two datasets were used. The outbreak dataset included 607 animals from one multiplier herd, whereas the gilt acclimation (GA) dataset included data on 2364 replacement gilts from seven breeding companies placed on health-challenged farms. Genomic prediction was evaluated using GA for training and validation, and using GA for training and outbreak for validation. Predictions were based on SNPs across the genome (SNP_All_), SNPs in one (SNP_MHC_ and SNP_130_) or both (SNP_SSC7_) QTL, or SNPs outside the QTL (SNP_Rest_).

**Results:**

Heritability of S/P in the GA dataset increased with the proportion of PRRS-positive animals in the herd (from 0.28 to 0.47). Genomic prediction accuracies ranged from low to moderate. Average accuracies were highest when using only the 269 SNPs in both QTL regions (SNP_SSC7_, with accuracies of 0.39 and 0.31 for outbreak and GA validation datasets, respectively. Average accuracies for SNP_ALL_, SNP_MHC_, SNP_130_, and SNP_Rest_ were, respectively, 0.26, 0.39, 0.21, and 0.05 for the outbreak, and 0.28, 0.25, 0.22, and 0.12, for the GA validation datasets.

**Conclusions:**

Moderate genomic prediction accuracies can be obtained for PRRS antibody response using SNPs located within two major QTL on SSC7, while the rest of the genome showed limited predictive ability. Results were obtained using data from multiple genetic sources and farms, which further strengthens these findings. Further research is needed to validate the use of S/P ratio as an indicator trait for reproductive performance during PRRS outbreaks.

**Electronic supplementary material:**

The online version of this article (doi:10.1186/s12711-016-0230-0) contains supplementary material, which is available to authorized users.

## Background

Porcine reproductive and respiratory syndrome (PRRS) is a major viral disease that impacts pork production worldwide [1] and results in decreased reproductive performance in sows [2, 3] and reduced growth performance in finisher pigs [4]. The economic impact that the PRRS virus (PRRSV) has on just the US swine industry is US$664 million per year, including breeding and growing pig herds, with an average loss of US$115 per breeding female [5].

Replacement gilts sourced from multiplier herds are usually introduced into commercial herds following acclimation and vaccination procedures that aim at exposing these naïve animals to pathogens (or antigens) that are common to the herd, such as the strains of PRRSV that are circulating in the herd. Due to the impact of PRRS on the swine industry, gilt replacement strategies have been developed with the objective of reducing the chances of introduction of new diseases in the herd or of infection of the replacement gilts [6]. These strategies include not obtaining animals from external sources (i.e. internal replacement), quarantine, and voluntary exposure of replacement gilts to the pathogens that are endemic to the herd [7]. A strategy that has not been explored to date is the identification of animals that have greater genetic potential to withstand pathogen challenge during the acclimation period. This strategy could be accomplished by assessing the immune response of animals across time, combined with high-density genotype data that could be used for genomic prediction, with the objective of genetically improving animals to obtain better performance during acclimation and in subsequent parities.

Recent studies on host responses to PRRSV indicate that selection for improved performance following PRRSV infection may be feasible for both sow reproduction [2, 3, 8] and growing pigs [9]. For reproduction, Serão et al. [3] reported moderate to low heritability estimates for reproductive performance during a PRRS outbreak, ranging from 0.06 for number of stillborn to 0.12 for number of born dead. In contrast, PRRSV sample-to-positive (S/P) ratio, a semi-quantification of PRRSV-specific immunoglobulin (Ig) type G (IgG; a major antibody produced by the humoral immune system), had a high heritability estimate (0.45) and a high positive genetic correlation with favorable reproductive performance during the PRRS outbreak (−0.72 ± 0.28 for number of stillborn and +0.73 ± 0.24 for number of born alive). These results suggest that S/P has the potential to be used as a genetic indicator trait to select replacement gilts with more favorable reproductive performance during a PRRS outbreak.

Using data from a PRRS outbreak in a multiplier herd, Serão et al. [3] also reported the detection of quantitative trait loci (QTL) for reproductive performance and PRRS antibody response during the PRRS outbreak. For number of stillborn piglets, they identified a QTL on *Sus scrofa* (SSC) 2 (between 32 and 25 Mb) that accounted for 11 % of the total genetic variance for all markers across the genome (TGVM). They also reported two major QTL on SSC7 for S/P, which accounted for 40 % of the TGVM. One of these QTL was located in the major histocompatibility complex (MHC) region, between 24 and 31 Mb, and accounted for ~25 % of the TGVM. The other QTL on SSC7 was located between 128 and 129 Mb and accounted for ~15 % of the TGVM. These two QTL for S/P on SSC7 were recently validated on an independent commercial dataset [10], which is part of the data used in this current study. Orrett et al. [8] also identified trends toward associations between SNPs on SSC7 and farrowing mortality during a PRRS outbreak, although not in the same regions as Serão et al. [3, 10].

Genomic prediction for response to disease is of great interest to the swine genetics industry because: (1) disease traits are generally not expressed in the nucleus populations that are used for selection since nucleus and multiplier herds must maintain a high health status, (2) in many commercial herds, breeders strive to maintain high health or vaccinate the animals to reduce the effects of disease challenges, thus available disease phenotypes are not reliable, and (3) recording of disease phenotypes can be expensive (e.g. measurement of antibody and viremia levels in blood).

Studies pertaining to the accuracy of genomic prediction of host response to PRRS are still very limited, and to date, only results using nursery piglets have been reported. Boddicker et al. [11], using data on ~1400 nursery piglets (initial age between 25 and 35 days) from different genetic suppliers and that were followed for 42 days after experimental infection with one isolate of type 2 PRRSV (NVSL 97-7985), reported moderate genomic prediction accuracies for viral load (measurement of total viral burden during the trial) and weight gain across cross-validation scenarios. These authors compared genomic prediction accuracies that were obtained by using only the SNPs within a QTL region on SSC4 that was previously identified for PRRS response [9] and by using SNPs within the rest of the genome (i.e. SNPs outside this QTL region). When the SNPs within this QTL region were used, average accuracies were equal to 0.34 and 0.48 for weight gain and viral load, respectively, whereas when SNPs within the rest of the genome were used, average accuracies of 0.21 and 0, for weight gain and viral load, respectively were obtained which indicated little to no predictive ability. Using the same data as Boddicker et al. [11] plus another ~1000 nursery piglets infected with a different strain of type 2 PRRSV (KS2006-72109), Waide et al. [12] compared the accuracy of genomic prediction when training was on response to one strain and validation on response to the other strain of the PRRSV. These authors reported similar accuracies for viral load between strains (~0.37), but observed a lower accuracy for weight gain when the training data were from animals infected with the KS06 strain (0.17) than with the NVSL strain (0.40).

The objectives of this study were to estimate genetic parameters for PRRSV antibody response during gilt acclimation in health-challenged farms, to identify regions associated with this response, and to assess the accuracy of genomic prediction of PRRSV antibody response in replacement gilts during acclimation and in sows following a reproductive PRRS outbreak. We compared the accuracy of genomic prediction of PRRSV antibody response using genotype data from the whole genome, by using only the SNPs that are located in the two QTL regions on SSC7 that were previously associated with PRRSV antibody response, and the SNPs from the rest of the genome. In addition, these analyses were performed in datasets with different proportions of PRRSV-seropositive animals in the herd.

## Methods

Animals used in this study were cared for according to Canadian Council on Animal Care [13] guidelines under standard industry conditions.

### Description of the datasets

The datasets used in this study were provided by a consortium of the main pig breeding companies (genetic suppliers) that operate in Canada (PigGen Canada, http://www.piggencanada.org/). The two datasets included data on (1) purebred multiplier gilts and sows and (2) commercial F1 replacement gilts. A detailed description of the first dataset is in Serão et al. [3]. Briefly, this dataset included high-density SNP genotype and phenotype data on 607 purebred Landrace gilts and sows from a commercial multiplier herd in Canada that experienced a PRRS outbreak that was estimated to have occurred on November 20th, 2011. Blood samples were collected on January 5th, 2012, and used for semi-quantification of PRRSV-specific IgG (measured as S/P ratio) by ELISA (IDEXX PRRS X3, IDEXX Laboratories Inc., Westbrook, ME, USA) and for genotyping using the Illumina PorcineSNP60BeadChip v.1 (Illumina Inc., San Diego). Thus, PRRS antibody levels (S/P ratio) were evaluated approximately 46 days after the outbreak. This dataset will hereafter be referred to as the outbreak dataset.

The second dataset, hereafter referred to as the gilt acclimation (GA) dataset, included data on naive crossbred (Landrace × Large White) replacement gilts sourced from different multiplier herds and genetic suppliers. Gilts were introduced into commercial herds that were pre-selected for this study based on historical occurrence of natural disease challenges, in groups of 10 to 63 animals (contemporary groups; CG), where they followed standard acclimation and gilt rearing procedures. Blood samples were collected on all gilts at the time of introduction into the commercial herd and at three subsequent time points: after the acclimation period, during parity 1, and during parity 2. Summary statistics on the number of multiplier herds, CG and farms, and PRRS vaccination use by farm are in Table 1.

**Table 1:** Summary statistics for contemporary groups by genetic supplier

GS (*n*)	MH	CH	CG (average $\bar n$)	PRRS Vx	Days (standard deviation)
1 (381)	1	1	3 (33.0)	Yes	88 (13.5)
	2	2	6 (20.2)	Yes	n/a
	3	3	4 (33.5)	Yes	72 (5.7)
		4	5 (22.0)	Yes	73.7 (3.8)
2 (277)	4	5	9 (13.3)	Yes	58 (21.8)
	5	6	4 (20.8)	No	29 (2.6)
	6	7	5 (18.0)	Yes	30.8 (2.8)
		8	4 (11.8)	Yes	32.8 (3.1)
3 (425)	7	9	3 (40.0)	Yes	35 (3.5)
	8	10	9 (46.3)	Yes	37.7 (4.2)
4 (368)	9	11	5 (18.4)	No	32.5 (2.4)
	10	12	4 (33.3)	Yes	38 (3.6)
		13	5 (30.0)	No	33.4 (3.0)
5 (367)	11	14	5 (26.2)	Yes	36.6 (1.1)
	12	15	4 (24.3)	No	49.7 (7.6)
	13	16	3 (33.7)	Yes	32 (5.3)
		17	4 (30.0)	Yes	33.7 (3.5)
6 (333)	14	18	7 (24.9)	Yes	29.9 (2.5)
		19	2 (25.0)	Yes	32.5 (2.1)
	15	20	3 (25.0)	Yes	35 (0)
		21	3 (24.7)	Yes	32.3 (3.0)
7 (204)	16	22	4 (37.8)	No	41.5 (11)
	17	23	4 (39.8)	Yes	34 (5.8)

Genetic supplier (GS; number of gilts within parenthesis), multiplier herd (MH), commercial herd (CH), number of contemporary groups (CG) and average number of gilts (average $\bar n$) per CG, use of vaccination for PRRS (PRRS Vx), and average days from introduction to blood sampling after acclimation (days) by CG

Seven breeding companies (genetic suppliers), which are all members of PigGen Canada, provided gilts for this study. Seventeen multiplier herds were sourced with two to three multipliers per genetic supplier. Gilts were placed in 23 pre-selected commercial herds (i.e. sow farms) across Canada, either directly or via a quarantine barn. A commercial production herd always received gilts from only one multiplier, while the same multiplier provided gilts to one or two commercial herds. One hundred and five CG were used in this study, prior to quality control. The number of CG per commercial herd ranged from 2 to 9, with the average number of gilts per CG ranging from 11.8 to 46.3 across the 23 herds.

Before starting this study, the veterinarians for the 23 commercial herds provided general management information, such as general production procedures, quarantine, and use of medications and vaccinations. Table 1 shows that 18 out of 23 farms indicated the use of vaccination against PRRS. The type of vaccination was not always identified, although several farms indicated the use of modified live PRRSV vaccines. Whenever timing was indicated (number of farms), vaccination occurred during entry (3), during quarantine (10), during acclimation (4), mid-lactation (1), after weaning (1), or at alternate parities (1). It should be noted that it was not certain that all the indicated procedures were consistently performed throughout the duration of the study. In addition, these procedures were completely confounded with farm, and therefore, inferences on the impact of PRRS vaccination will be limited and should be interpreted with caution.

### Phenotypic data

Phenotypic data were collected on 2852 replacement gilts in the GA study. Similar to the outbreak dataset, blood samples were collected and used for semi-quantification of PRRSV-specific IgG [measured as sample-to-positive (S/P) ratio] by ELISA (IDEXX PRRS X3, IDEXX Laboratories Inc., Westbrook) at GREMIP (Université de Montréal, Montreal) and for genotyping with the Illumina PorcineSNP BeadChip (Illumina Inc., San Diego; see below) at Delta Genomics (Livestock Gentec, Edmonton). S/P was measured at four time points: at entry (S/P_Entry_), after the acclimation period (S/P_Post-acclimation_), during first parity (S/P_Parity1_), and during second parity (S/P_Parity2_). The exact sampling times were completely confounded with farm and not all sampling points were available for all farms. Collection dates were available for S/P_Entry_ and S/P_Post-Acclimation_ for all but one herd and were complete for 92 of 105 CG. Information on the average time interval between S/P_Entry_ and S/P_Post-Acclimation_ by herd is in Table 1 and ranged from 29 to 88 days, with an overall average of 40.8 ± 16.3 days. Intervals by CG ranged from 26 to 103 days. Collection dates were not available for S/P_Parity1_ and S/P_Parity2_, other than that these collections occurred some time between farrowing and weaning.

Preliminary analyses revealed that S/P_Entry_, S/P_Parity1_, and S/P_Parity2_ had low heritability estimates across the different S/P datasets (described below), ranging from 0 to 0.07. These estimates were usually combined with large standard errors and sometimes could not be estimated due to very low genetic variance. Therefore, only analysis of S/P_Post-acclimation_ will be described in the remainder of this paper, which hereafter will be referred to simply as S/P.

### Percentage of PRRSV-seropositive animals by contemporary group

Five S/P ratio GA datasets were created based on the percentage of animals with a positive (S/P ≥ 0.4) PRRSV ELISA test within a CG, with the objective of assessing its impact on heritability estimates and genomic prediction accuracies. Thresholds used to create the datasets were: ≥0, ≥25, ≥50, ≥75, and 100 % positive gilts within a CG, which will hereafter be referred to as the S/P_0%_, S/P_25%_, S/P_50%_, S/P_75%_, and S/P_100%_ datasets, respectively. Only CG with at least 10 animals were used for these calculations and further analyses (two CG were excluded). The numbers of animals, CG, commercial herds, and multipliers, as well as the percentage of PRRSV-seropositive animals across S/P datasets are in Table 2 and for S/P_0%_, were equal to 2346, 95, 23, and 17, respectively, and for S/P_100%_ to 1361, 56, 20, and 14, respectively. Data from all seven genetic suppliers were included in each S/P dataset.

**Table 2:** Summary statistics and genetic parameters for S/P^a^ ratio across the gilt acclimation and outbreak datasets

Item	Gilt acclimation dataset^b^					Outbreak dataset
	S/P_0%_	S/P_25%_	S/P_50%_	S/P_75%_	S/P_100%_	
Mean	1.19	1.36	1.40	1.45	1.55	1.79
Standard deviation	0.72	0.61	0.57	0.53	0.48	0.38
Seropositive rate (%)	81.0	92.3	95.4	97.9	100.0	100.0
Number of						
Animals	2364	2073	1969	1849	1361	607
Contemporary groups	95	83	79	73	56	1
Commercial herds	23	21	21	20	20	1
Multiplier herds	17	15	15	14	14	1
Genetic suppliers	7	7	7	7	7	1
Heritability	0.275	0.297	0.375	0.449	0.474	0.536
Standard error	0.041	0.045	0.046	0.047	0.057	0.110
Genetic variance	0.053	0.066	0.079	0.090	0.086	0.078
Residual variance	0.141	0.156	0.131	0.111	0.095	0.067

^a^Sample-to-positive ratio

^b^Subscripts for each S/P dataset represent the minimum percentage of PRRSV-seropositive animals within contemporary group

### Genotype data

A total of 3615 animals were genotyped using the Illumina PorcineSNP BeadChip and 48, 1710, and 1857 of these animals were genotyped using versions 60 K v.2, 60 K v.2B, and 80 K, respectively (Illumina Inc., San Diego). These versions include 62,163, 61,565, and 68,528 SNPs, respectively. A total of 42,145 SNPs that were common to all three versions were used for subsequent analyses.

Before analyses, the genotypes of the GA dataset were evaluated for quality. First, genotypes with a GenCall score lower than 0.5 were set to missing (5.25 %). Second, SNPs that had less than 80 % of genotypes called across all individuals (3954 SNPs) were excluded from the dataset. The final dataset included 38,191 SNPs for 3615 individuals and had a genotype call rate of 99.48 %. The genotype data of the outbreak dataset were filtered in order to include the same 38,191 SNPs.

Missing genotypes were replaced with the mean coded (0/1/2 reference alleles) of the SNP genotype within a multiplier to avoid problems with the statistical methods used (see below). For SNPs that were completely missing within a multiplier, the mean genotype was calculated using all individuals provided by the genetic supplier for that multiplier. No SNP was completely missing within genetic supplier.

Of the 3615 genotyped animals, 2947 were gilts and 668 were parents of the gilts. Although we had neither the phenotypes on the parents, nor pedigree information on the gilts to identify their parents, we kept the parental genotypes in the dataset to make use of their genomic relationships. Visual inspection of the genomic relationships showed inconsistent relationships of some animals with others from the same multiplier and/or genetic supplier. For instance, 80 animals showed genomic relationship coefficients lower than 0.03 with more than half of the animals from the same multiplier herd, and thus, were excluded, which resulted in 2867 gilts with genotype data. However, due to missing phenotypic data for S/P (S/P_Post-acclimation_) on 503 gilts, exclusion of another nine gilts based on the number of animals within CG and of another nine due to missing genotype data, final analyses were performed using 2346 gilts with phenotypes and genotypes.

### Genomic relationship matrix and genetic parameters

A genomic relationship matrix (GRM) was estimated for the GA dataset as proposed by VanRaden [14], using 38,191 SNPs and 3535 individuals (668 parents without phenotypes and 2867 gilts with or without phenotypes). Genotypes were coded as 0/1/2 and averaged and centered within multiplier herd. Relationships across multipliers, and therefore across genetic suppliers, were allowed since animals used in this study had a similar breed composition.

Using the GRM, the following genomic model was used to estimate variances and genetic parameters with ASReml 4.0 [15]:

$$y_{ij} = \mu + CG_{i} + u_{ij} + e_{ij} ,$$

where *y*_*ij*_ is the observed phenotype of individual *j* at the *i*th level of *CG*_*i*_, *CG*_*i*_ is the *i*th level of the fixed-effect of contemporary group, *u*_*ij*_ is the breeding value, and *e*_*ij*_ is the random error. Vectors of breeding values and residual effects were assumed to be normally distributed as: ${\mathbf{u}}\sim{\text{N}}\left( {0,{\mathbf{GRM}}\sigma_{u}^{2} } \right)$ and ${\mathbf{e}}\sim{\text{N}}\left( {0,{\mathbf{I}}\sigma_{e}^{2} } \right)$. Variance components for S/P were estimated for each of the five S/P datasets. The outbreak dataset had its own GRM, and genetic parameters for S/P in this dataset were estimated using the model described in Serão et al. [3].

### Genome-wide association

Bayesian genomic prediction methods were used to perform a genome-wide association study (GWAS) for S/P, using GenSel version 4.4 [16] ratio. In addition to fitting SNP effects as random effects, the GWAS model included the same fixed effects as used for estimation of genetic parameters, with estimates of additive genetic and residual variances obtained from that model as priors. Bayesian method C*π* [17] was used to estimate the proportion of SNPs with zero effects (*π* = 0.987) and Bayes-B with this estimate of *π* was used for the GWAS, consistent with the original analysis of Serão et al. [3].

### Genomic prediction analyses

Bayesian genomic prediction methods were used to estimate the effects of SNPs in the training dataset, using the same models as used for GWAS in GenSel version 4.4 [16]. Bayesian C*π* [17] was used to estimate the proportion of SNPs with zero effects (*π*) for each training dataset. Three genomic prediction methods were used for training, with two methods based on the estimated π (Bayes-B and Bayes-C) and one using *π* = 0 (Bayes-C0; hereafter referred to as GBLUP). Estimates of *π* were similar across training datasets, ranging from 0.987 to 0.991.

Estimates of SNP effects for S/P were obtained by including all SNPs in the training analyses. Based on the initial findings of Serão et al. [3], five sets of SNPs were then used for genomic prediction:

1. All SNPs across the genome (SNP_All_).
2. SNPs in the QTL_MHC_ region (SNP_MHC_).
3. SNPs in the QTL_130_ region (SNP_130_).
4. The combination of SNPs in the QTL_MHC_ and QTL_130_ regions (SNP_SSC7_).
5. All SNPs across the genome excluding those in the QTL_MHC_ and QTL_130_ regions (SNP_Rest_).

Although Serão et al. [3] reported that QTL_MHC_ and QTL_130_ were located within the regions between 24 and 30 Mb and between 128 and 129 Mb on SSC7, respectively, the QTL intervals that were used herein were obtained from the GWAS of each of the five S/P datasets. The QTL intervals were defined using the two outermost 1-Mb SNP windows that explained at least 1 % of TGVM. Within the outermost 1-Mb windows, the SNP that had the highest posterior probability of inclusion (PPI) [18] was identified and the QTL region was further extended by 2 Mb to account for the limited resolution of the GWAS methods that were used [18] and to remove any QTL signal when using the regions outside the QTL interval. The QTL intervals were similar for the five S/P datasets (data not shown) and thus, for simplicity, we used the QTL limits derived from the S/P_0%_ analyses (see Table 3).

**Table 3:** Sample sizes of the gilt acclimation training datasets and of the corresponding validation datasets used for genomic prediction validation analyses

Validation scenario	Gilt acclimation datasets^a^					Validation data (GS^b^)
	S/P_0%_	S/P_25%_	S/P_50%_	S/P_75%_	S/P_100%_	
7-fold cross-validation						
Fold 1	1983	1712	1647	1588	1149	212 (GS 1)
Fold 2	2078	1788	1684	1587	1201	160 (GS 2)
Fold 3	1939	1696	1592	1508	1233	128 (GS 3)
Fold 4	1996	1761	1722	1602	1114	247 (GS 4)
Fold 5	1997	1759	1655	1535	1101	260 (GS 5)
Fold 6	2031	1740	1636	1516	1028	333 (GS 6)
Fold 7	2160	1982	1878	1758	1340	21 (GS 7)
Outbreak dataset	1939	1696	1592	1508	1233	607

^a^Subscript values for each S/P dataset represent the minimum percentage of PRRSV-seropositive animals within contemporary group

^b^ *GS* Genetic supplier

### Training and validation datasets

To assess the accuracy of S/P predictions, two training and validation scenarios were used.

#### Genomic prediction in the outbreak dataset

The GA dataset was used for training and the outbreak dataset was used for validation. Genotypes of 425 individuals that were from the same genetic supplier as the outbreak dataset were excluded from the training (GA) dataset for this analysis, which will hereafter be referred to as the reduced-GA dataset and included 1939 animals from six genetic suppliers. Independence of the outbreak and reduced-GA datasets was assessed by principal component analysis of the genotype data.

#### Sevenfold cross-validation for the GA dataset

Data from six of the seven genetic suppliers were used as the training dataset and data from the other genetic supplier was used as the validation dataset. This was repeated until all seven genetic suppliers were used as the validation dataset. For validation, only CG included in the S/P_100%_ dataset were used in order to allow comparison to accuracies obtained in scenario 1, since all animals in the outbreak dataset were also tested positive. Preliminary analyses showed that using S/P_100%_ for validation yielded slightly better and more consistent results across SNP sets than other S/P datasets.

The numbers of individuals used for training and validation are in Table 3.

Population structure of the GA dataset was evaluated by constructing a neighbor joining [19] phylogenetic tree based on Nei’s genetic distance [20]. Genetic distances between genetic suppliers were calculated using the R package StAMPP [21] and plotted using MEGA 6 software [22]. Only animals used for genomic prediction (i.e. with phenotype and genotype data) were used in this step. Population structure was evaluated for each SNP set.

### Accuracy of genomic prediction

When the complete GA dataset was used for training and the outbreak dataset for validation, genomic prediction accuracy was estimated as:

$$Accuracy = \frac{{r_{{\left( {GEBV,y^{*} } \right)}} }}{{\sqrt {h^{2} } }},$$

where $r_{{\left( {GEBV,y^{*} } \right)}}$ is the correlation of the genomic estimated breeding values (*GEBV*) with phenotypes from the outbreak dataset adjusted for estimates of fixed-effects ($y^{*}$) [3], and *h*^2^ is the marker-based heritability in the outbreak dataset.

For the sevenfold cross-validation in the GA dataset, accuracy of genomic prediction was calculated as a weighted average correlation across validation sets:

$$Accuracy = \frac{{\mathop \sum \nolimits_{i = 1}^{7} n_{i} r_{{i} \left( {GEBV,y^{*} }\right)}}}{{\frac{{\mathop \sum \nolimits_{i = 1}^{7} n_{i} }}{{\sqrt {h^{2} } }}}},$$

where $r_{{i\left( {GEBV,y^{*} } \right)}}$ and *n*_*i*_ are the correlation of *GEBV* with $y^{*}$, and the number of animals, respectively, in the *i*th genetic supplier validation dataset. Phenotypes were adjusted for estimates of the fixed effect of CG obtained from the validation dataset only, using a model that included only CG as fixed effect. The marker-based heritability (*h*^2^) used in this step was from the whole S/P_100%_ GA dataset (Table 2).

## Results

### Genetic parameters

Estimates of genetic parameters for S/P are in Table 2. Heritability estimates in the GA dataset increased as the percentage of PRRS ELISA-positive animals within CG increased, from 0.28 ± 0.04 for S/P_0%_ to 0.47 ± 0.06 for S/P_100%_. Increases in heritability estimates were due to increasing estimates of genetic variance, from 0.05 for S/P_0%_ to 0.09 for S/P_100%_, and decreasing estimates of residual variance as the percentage of PRRSV-seropositive animals increased. The heritability estimate for S/P in the outbreak dataset (0.54 ± 0.11) was slightly higher than that for the GA dataset with 100 % PRRSV-seropositive animals, primarily because of a lower residual variance estimate.

### GWAS results and location of the two QTL

**Fig. 1:**
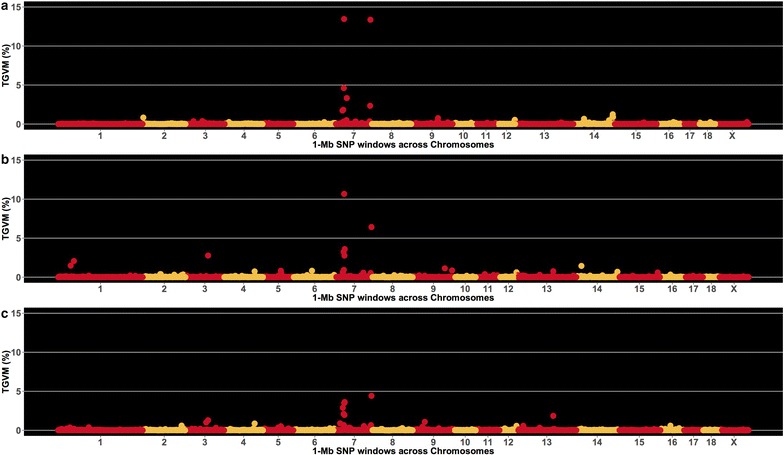
Manhattan plot for sample-to-positive (S/P) ratio. Each data point represents a 1-Mb SNP window plotted against the proportion of total genetic variance accounted for by the markers (TGVM). The chromosomes (1 to 18, and X) and SNPs are ordered from *left* to *right*. Plots **a**, **b**, and **c** represent results for the original findings by Serão et al. [3] using the outbreak dataset, and for the gilt acclimation (GA) dataset using S/P_0**%**_ and S/P_100**%**_, respectively

Results from GWAS using the GA dataset and the original analysis of the outbreak dataset of Serão et al. [3] are in Fig. 1a. The two QTL on SSC7 for S/P that were originally reported by Serão et al. [3] for the outbreak dataset were also identified in the GA dataset for S/P_0%_ (Fig. 1b) and S/P_100%_ (Fig. 1c). The S/P_100%_ data showed a lower signal at QTL_MHC_ and QTL_130_ than the S/P_0%_ and the outbreak data. Results for S/P_25%_, S/P_50%_ and S/P_75%_ (not shown) were similar to those for S/P_0%_.

Locations of the two QTL on SSC7, using S/P_0%_, are in Table 4. In the GA data, QTL_MHC_ and QTL_130_ accounted for 20.1 and 6.7 % of the TGVM, respectively, for S/P_0%_ (Fig. 1b), and 15.2 and 4.7 % of the TGVM, respectively, for S/P_100%_ (Fig. 1c). In the outbreak dataset, these two QTL accounted for 25.2 and 15.7 % of the TGVM, respectively (Fig. 1a).

**Table 4:** QTL intervals^a^ and number of SNPs for each SNP set used for genomic prediction^b^

SNP dataset	QTL interval (Mb)	Number of SNPs
SNP_All_		38,191
SNP_MHC_	ALGA0039404 (22.9) to ASGA0032334 (33.5)	175
SNP_130_	ALGA0045559 (127.9) to ALGA0045891 (132.5)	94
SNP_SSC7_		269
SNP_Rest_		37,922

^a^Intervals for chromosome 7 only

^b^Determined using the gilt acclimation (GA) dataset

### Genomic prediction

#### Population structure

**Fig. 2:**
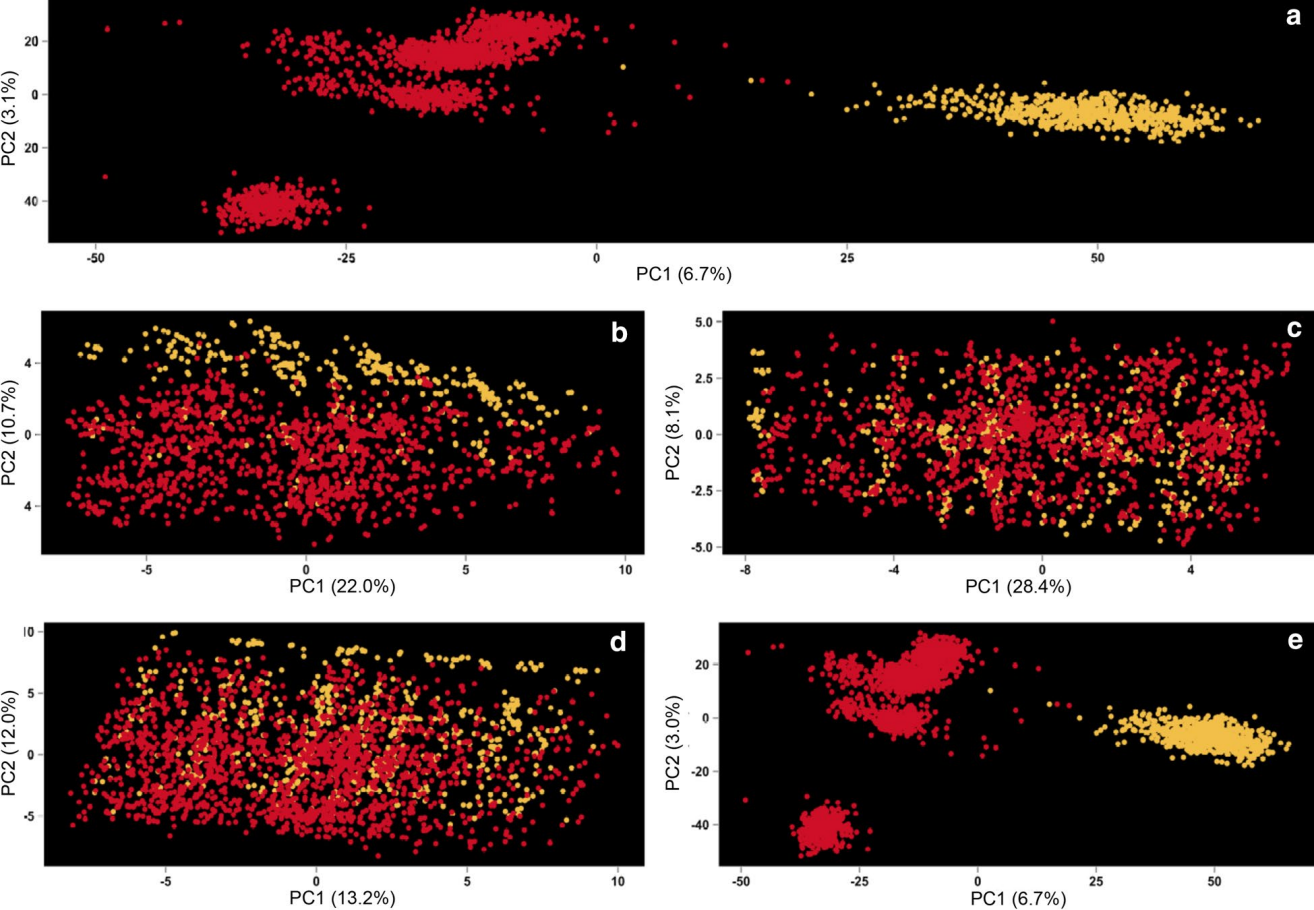
Population structure between the reduced-gilt acclimation and outbreak datasets. Plots of the first two principal component scores (PC1 and PC2) generated from SNP genotypes included in the five SNP datasets. *Each dot* represents one animal. *Red dots* represent animals from the reduced-gilt acclimation and *yellow dots* represent animals from the outbreak dataset. PC score plots in **a**, **b**, **c**, **d**, and **e** are based on all SNPs across the genome (SNP_ALL_), SNPs in QTL_MHC_ (SNP_MHC_), SNPs in QTL_130_ (SNP_130_), SNPs in both QTL (SNP_SSC7_), and SNPs outside both QTL (SNP_Rest_), respectively

A principal component analysis was performed to assess differences in the genetic background between the outbreak (validation) and reduced-GA (training) datasets (Fig. 2). The first principal component (PC1) explained 6.7, 22.0, 28.4, 13.2, and 6.7 % of the total variance for SNP sets SNP_All_, SNP_MHC_, SNP_130_, SNP_SSC7_, and SNP_Rest_, respectively, whereas the second principle component explained 3.0, 10.7, 8.1, 12.0, and 3.0 % of the total variance, respectively. Figure 2a (SNP_All_) and e (SNP_Rest_) demonstrate that the reduced-GA and outbreak datasets were genetically independent, with the two groups clustering in different areas of the plot. When using the other SNP sets (Fig. 2b–d), the reduced-GA and outbreak datasets were not well discriminated, although some discrimination could be observed for PC2 when using SNPs in the MHC region (SNP_MHC_; Fig. 2b).

**Fig. 3:**
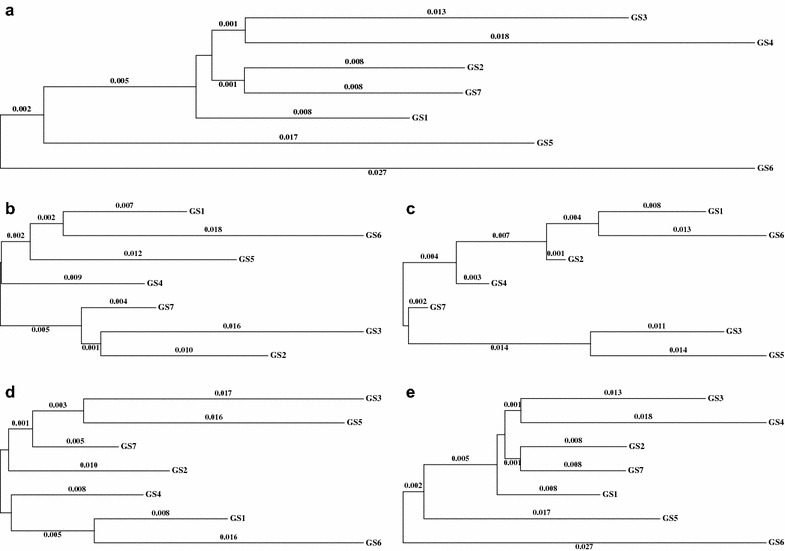
Population structure between the seven genetic suppliers of the gilt acclimation datasets. Genomic distances between genetic suppliers (GS) were estimated for each SNP dataset. Numbers on each branch represent Nei’s genetic distance between the taxon and the node. Neighbor joining trees in **a**, **b**, **c**, **d**, and **e** are based on all SNPs across the genome (SNP_ALL_), SNPs in QTL_MHC_ (SNP_MHC_), SNPs in QTL_130_ (SNP_130_), SNPs in both QTL (SNP_SSC7_), and SNPs outside both QTL (SNP_Rest_), respectively. Values for genetic distances less than 0.001 are not shown

Since the GA dataset included data from seven genetic suppliers, we assessed differences in genetic background between genetic suppliers based on their genetic distances for each of the five SNP sets (Fig. 3). Results for SNP_All_ (Fig. 3a) and SNP_Rest_ (Fig. 3e) were the same, with genetic supplier 6 (GS 6) branching separately (i.e. genetically diverging) from the other GS. Genetic distances based on the QTL SNPs (Fig. 3b–d) differed from those based on SNP_All_ and SNP_Rest_. Overall, genetic distances based on SNP_All_ and SNP_Rest_ were larger (average distance of 0.01) than those based on QTL SNPs, i.e. 0.008, 0.007, and 0.008 for SNP_MHC_, SNP_130_, and SNP_SSC7_, respectively.

#### Genomic prediction accuracies

**Fig. 4:**
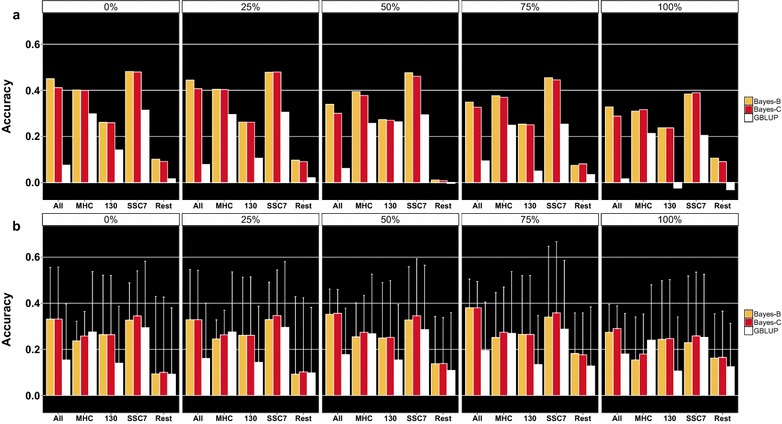
Genomic prediction accuracies for three methods and for different validation, SNP, and sample-to-positive (S/P) datasets. Genomic prediction accuracies in the outbreak and gilt acclimation (GA) datasets are in **a** and **b**, respectively. Results when using S/P datasets from S/P_0%_ to S/P_100%_ used for training are in panels designated by 0 to 100 %, respectively. Within each panel, color-coded bars represent genomic prediction accuracies for each method across SNP datasets. SNP datasets SNP_All_, SNP_MHC_, SNP_130_, SNP_SSC7_, and SNP_Rest_ are represented by All, MHC, 130, SSC7, and Rest, respectively. For the GA validation results (**b**), *white error bars* represent the standard deviation of accuracies across the seven cross-validation folds

Genomic prediction accuracies for the outbreak (Fig. 4a) and GA (Fig. 4b) validation datasets generally showed consistency across genomic prediction methods, SNP sets, and S/P training datasets (see Additional file 1: Table S1). Results for each fold when training and validating using the GA data are presented in Figures S1, S2, S3, S4, S5, S6 and S7 (see Additional file 2: Figures S1, S2, S3, S4, S5, S6 and S7). Overall, accuracies were higher for the outbreak than for the GA validation datasets. For the outbreak validation dataset, SNP_SSC7_ resulted in the highest accuracy, followed by SNP_MHC_, SNP_All_, SNP_130_, and SNP_Rest_, with overall average accuracies across methods and S/P datasets of 0.39, 0.34, 0.26, 0.21, and 0.05, respectively. For the GA validation dataset, SNP_SSC7_ resulted in the highest accuracy, followed by SNP_All_, SNP_MHC_, SNP_130_, and SNP_Rest_, with overall average accuracies of 0.31, 0.28, 0.25, 0.22, and 0.12, respectively. In general, Bayesian model selection methods (Bayes-B and Bayes-C) showed higher accuracies than GBLUP. This was always the case when the outbreak dataset was used for validation, but GBLUP showed higher accuracies than Bayes-B and Bayes-C with the GA validation dataset for SNP_MHC_ (S/P_0%_, S/P_25%_, and S/P_100%_).

Increasing the percentage of PRRSV-seropositive animals in the GA dataset used for training had opposite effects on accuracies of prediction in the outbreak and GA datasets. When the outbreak dataset was used for validation, there was a steady decrease in accuracy as the percentage of PRRSV-seropositive animals increased from S/P_0%_ to S/P_75%_. However, this decrease in accuracy was greater when using SNP_All_ than when using QTL SNPs. In contrast, when performing cross-validation with the GA dataset, there was an increase in genomic prediction accuracies from S/P_0%_ to S/P_75%_.

## Discussion

This study analyzed data from commercial herds to (1) identify and validate genomic regions associated with PRRSV ELISA antibody response, measured as S/P ratio; (2) evaluate the use of different SNP sets, with or without QTL SNPs, for genomic prediction of S/P in commercial gilts and sows; and (3) assess the impact of the proportion of PRRSV-seropositive animals on heritability estimates and accuracy of genomic prediction.

Serão et al. [3] performed a GWAS for S/P in a reproductive PRRS outbreak herd and identified two QTL on SSC7 that together accounted for ~40 % of the TGVM. These QTL were validated by Serão et al. [10] using part of the GA data reported herein. One of these QTL spans the MHC region (QTL_MHC_), which harbors genes associated with immune response to infectious diseases and vaccines [23], and explained ~25 % of the TGVM [3]. Associations between high-density SNPs in the MHC region have also recently been reported with porcine circovirus type-2b serum viremia in pigs [24, 25], PRRS antibody response in pigs [26], and with immune response indicators in dairy cattle [27]. The other QTL, QTL_130_, explained ~15 % of the TGVM [3] and was located approximately 100 Mb downstream of QTL_MHC_. Serão et al. [3] also reported candidate genes in this region, bringing special attention to the *tumor necrosis factor receptor*-*associated factor 3* gene (*TRAF3*), which has an important role in immune response activation [28]. Due to the importance that these regions have for immune response, we specifically evaluated the predictive ability of these two QTL on SSC7 for PRRSV antibody response, compared to the whole genome or the rest of the genome.

The PRRSV antibody response data used in this study were collected on commercial animals sourced from different genetic suppliers and reared under standard production settings in commercial herds. The use of field data has several advantages, such as working with a large sample size, use of animals from different genetic backgrounds (genetic suppliers and multipliers) that represent current commercial crossbred genetics, and the use of different environments under a range of disease pressures and current industry production practices. However, the main disadvantages of using field data are the variability in management and data collection procedures between farms, such as the use and timing of vaccination, inability to account for multiple unidentified sources of variation, and limitations in obtaining additional information that could improve the analyses, such as age of the animals and complete pedigree. However, in spite of the heterogeneity of the GA dataset, we obtained several promising results, including moderate to high heritabilities of PRRSV antibody response of replacement gilts, measured as S/P after the acclimation period, and low to moderate-high accuracies of genomic prediction of antibody response following a reproductive PRRS outbreak based on S/P following acclimation, especially when using SNPs located within QTL_MHC_ and QTL_130_. In both the GA and outbreak datasets, S/P ratio was measured, on average, around peak PRRS antibody response (40 to 60 days after exposure), although the time of measurement varied greatly in the GA data (from 26 to 103 days) following introduction into the herd. Additional studies are necessary to evaluate the impact of the time of S/P ratio measurement and of method of exposure on genomic prediction accuracies.

Another point that requires attention is the fact that none of the animals used in this study were experimentally infected with PRRSV. In the outbreak dataset, all animals were naturally infected with PRRSV and infection likely occurred within a limited amount of time. In contrast, in the GA dataset, pigs within a CG were exposed using different methods, including vaccination with a modified live PRRSV, natural exposure, or deliberate exposure using other acclimation methods, but the specific method used for each CG was uncertain. In addition, the timing of the outbreak or vaccination was variable and often unknown. Nevertheless, it is known and expected that natural PRRSV infection and response to modified live PRRSV vaccination result in similar antibody responses [29]. Nevertheless, the moderate to high heritabilities of S/P in both the GA and outbreak data and moderate accuracies of genomic prediction of S/P in the outbreak data based on training in the GA data suggest that antibody responses to vaccination or infection with PRRSV are similar traits and have the potential to be interchangeably used for genomic prediction of PRRS ELISA S/P ratio.

### Genetic parameters for S/P ratio

Estimated heritability of S/P was numerically lower in the GA datasets (0.28 to 0.47) than in the outbreak dataset (0.54). This is likely due to the greater uniformity of the data in the outbreak herd. For example, all animals in the outbreak dataset were from the same genetic source and farm. In addition, the outbreak dataset was composed of animals that underwent a PRRS outbreak during the same period of time. These more homogeneous environmental conditions were reflected in the lower residual variance for the outbreak dataset (from 0.07 vs. 0.10 to 0.16 for the GA datasets). However, the marker-based heritability (0.54) reported here for the outbreak dataset was greater than the pedigree-based estimate (0.45) reported by Serão et al. [3] for the same dataset. Although heritability estimates for S/P were lower with the GA dataset than with the outbreak dataset, they were moderate to high, in spite of the heterogeneity of these field data.

In general, the estimate of residual variance for S/P decreased and the estimate of genetic variance increased as the percentage of positive animals for the PRRSV ELISA test within CG increased, thereby increasing heritability estimates (Table 2). The impact of disease prevalence on heritability estimates from field data was well addressed by Bishop and Woolliams [30]. These authors showed that the true heritability is underestimated when exposure to infection is incomplete. In other words, heritability estimates are expected to increase as disease prevalence, or the proportion of animals identified as diseased, increases. In our study we could not distinguish PRRSV antibody response (S/P ratio) resulting from PRRSV infection from response to PRRSV vaccination, thus we could not confirm that the increase in heritability was only due to prevalence. Nevertheless, this same pattern was observed in our study, with higher heritabilities in datasets with a larger number of positive animals.

### GWAS results and location of the two QTL

The locations of the QTL identified in this study encompassed the two regions that were originally reported by Serão et al. [3], using the outbreak data. QTL_MHC_ and QTL_130_ were positioned between 22.9 and 33.5 Mb and between 127.9 and 132.5 Mb on SSC7, respectively, compared to between 24.0 and 30.9 Mb and between 128.0 and 129.9 Mb in the previous study of Serão et al. [3]. Since the objective of this study was to evaluate the use of SNPs within these QTL for genomic prediction, we further increased the regions by 2 Mb based on the furthermost SNPs that had the highest probability of inclusion within a 1-Mb windows that explained at least 1 % of the TGVM.

Overall, the GWAS results were consistent across S/P_0%_ to S/P_75%_. QTL_MHC_ had similar %TGVM in the GA dataset (~20 %) as in the outbreak data (~25 % [3]). Results for QTL_130_ were lower in the GA dataset (S/P_0%_ to S/P_75%_) than in the outbreak dataset, with ~7 and ~15 % of the TGVM, respectively. For S/P_100%_, the %TGVM explained by these QTL was slightly lower (15.2 % for QTL_MHC_ and 4.7 % for QTL_130_) than for S/P_0%_ to S/P_75%_. These differences could be due to the big drop in sample size from S/P_75%_ (*n* = 1849) to S/P_100%_ (*n* = 1361). Nevertheless, these results are promising, considering that the outbreak data included data from only one herd and genetic line, while the GA data included data from multiple genetic suppliers, multipliers and commercial herds.

### Genomic prediction

#### Population structure

The principal component analysis using SNP_All_ (Fig. 2a) indicated that the reduced-GA training population was genetically independent of the outbreak population. In contrast, this genetic independence between datasets was not so clear for the PCA plots that were based only on the QTL SNPs. For SNP_MHC_ (Fig. 2b), there was no discrimination between the outbreak and reduced-GA datasets based on PC1 (22.0 %) but there was some discrimination based on PC2 (10.7 %). This lack of discrimination could be due to the high level of polymorphism observed in the MHC region. Overall, there was considerable overlap between PC scores from the two datasets when QTL SNP sets were used.

For the GA datasets, we assessed differences in genetic background by visualizing trees based on Nei’s genetic distance. Similar to what was observed based on the PC analysis of the outbreak and reduced-GA datasets, SNP_SSC7_ (Fig. 3d) showed a combination of the results for SNP_MHC_ and SNP_130_ (Fig. 3b, c, respectively). However, the two main clades for SNP_MHC_ and SNP_130_ included different genetic suppliers, indicating that the genetic diversities represented by these two QTL are in part independent from each other. As expected, the trees based on SNP_All_ and SNP_Rest_ were the same, which indicates that the SNP QTL do not play a major role in assessing the overall genetic distance between genetic suppliers.

Thus, both methods used to assess within- and across-population diversity showed that SNP_All_ and SNP_Rest_ discriminated populations well, while SNPs on both QTL (SNP_MHC_, SNP_130_, and SNP_SSC7_) showed considerable relationships across populations, suggesting that these sets of SNPs should indeed result in higher across-population genomic prediction accuracies.

#### Genomic prediction accuracies

Reports on genomic prediction of antibody response are scarce in the literature. In poultry, Liu et al. [31] evaluated the use of high-density SNP genotypes for prediction of antibody response to Newcastle disease and avian influenza across scenarios with different levels of relationships between the training and validation datasets. With lower relationships between datasets, they observed moderate to high genomic prediction accuracies for antibody response to both diseases, ranging from 0.30 to 0.61, after dividing their reported correlations of predictions with phenotype by the square root of the marker-based heritability (for proper comparison with our study). Accuracies of genomic prediction were higher when greater relationships between training and validation were allowed (0.46 to 0.74). In our study, we were not interested in within-population predictions but in the use of the major QTL for S/P for prediction, regardless of the relationships between populations.

Overall, the accuracies of genomic prediction for PRRS S/P ratio that we obtained in this study ranged from low (typically when using SNP_Rest_) to moderate-high (mostly with SNP_SSC7_, SNP_MHC_ and SNP_All_), which suggested that the rest of the genome had little predictive ability and that only SNPs within the two QTL (QTL_MHC_ and QTL_130_) were needed to obtain sizeable genomic prediction accuracies across genetically different populations. Although accuracies differed between folds (i.e. genetic suppliers), S/P datasets, and SNP sets when using the GA dataset (cross-validation), the differences in accuracies when validation was done with the GA versus the outbreak dataset were interesting; while there was a clear increase in genomic prediction accuracies as the percentage of PRRSV-seropositive animals in the training dataset increased (S/P_0%_ versus S/P_100%_) when validation was done with the GA dataset, accuracies decreased from S/P_0%_ to S/P_100%_ when using the outbreak data for validation. Based on the increase in heritability with increasing % of positive animals in the GA training dataset, one would expect accuracies to increase with % of positive animals. However, the increase in % of positive animals in the GA training data was confounded with a decrease in the size of the training data, which is expected to decrease accuracies. In order to assess the effect of sample size on genomic prediction accuracies using the outbreak validation dataset, we randomly selected CG from the reduced-GA training dataset to have a similar number of animals as in the S/P_100%_ dataset (*n* = 1233) and a similar percentage of PRRSV-seropositive animals in S/P_0%_ (81 %). We performed five replicates and results yielded similar accuracies (e.g. average accuracy of 0.323 using Bayes-B) as obtained when using S/P_100%_ for training and validating with the outbreak data. Thus, the drop in accuracies with the outbreak dataset from training on S/P_0%_ to S/P_100%_ was due to the smaller number of animals used for training, and not due to differences in the % of positive animals.

However this does not explain the increase in accuracy with % of positive animals that was observed for the GA validation data. There are several differences between the GA and outbreak validation datasets that may have contributed to these opposite trends in accuracies. For example, although “noisy”, the data from different herds within the GA dataset are expected to be more similar to each other than to the outbreak dataset, since the GA dataset included data from several commercial production herds, while the outbreak dataset was composed of data from a more controlled environment (i.e. a single commercial multiplier herd). In addition, the impact of sample size and % of PRRSV-seropositive animals on the accuracy of genomic prediction could also be a factor explaining the differences in results between validation datasets. When validation was done with the outbreak dataset, a larger training population size (S/P_0%_, *n* = 1939 vs. e.g. S/P_100%_, *n* = 1233) was needed to achieve the highest accuracy, while with the GA dataset, the largest training population size (S/P_0%_; average *n* = 2023) resulted in lower accuracies.

Another component that could be added to this discussion is that the GA dataset consisted of antibody responses both to vaccination and natural exposure, whereas the outbreak dataset was the result of natural exposure only. Although reports indicate that, phenotypically, animals that are infected with PRRSV have similar antibody responses to those that are vaccinated with a modified live vaccine, this may not be true at the genetic level. In addition, there are likely to be some differences between the modified live strain of PRRSV and the strain of virus responsible for the outbreak that may influence the effect of host genetics.

Genomic prediction accuracies obtained across all methods and validation and S/P datasets suggest that the effects of the two major QTL on SSC7 for S/P are additive and, thus, orthogonal to each other; accuracies using SNP_SSC7_ could be approximately derived as the square of the sum of squared accuracies from using SNP_MHC_ and SNP_130_. For example, using the average accuracies with SNP_MHC_ and SNP_130_ in the GA dataset (0.25 and 0.21, respectively), the accuracy based on SNP_SSC7_ based on additivity is expected to be $\sqrt {0.25^{2} + 0.21^{2} }$ = 0.33, which is approximately the average accuracy obtained using SNP_SSC7_ (0.31) across all methods and S/P datasets. This was also observed for the outbreak dataset: $\sqrt {0.34^{2} + 0.21^{2} }$ = 0.40 ≈ 0.39, the average accuracy across methods and S/P datasets using SNP_SSC7_. These results also showed that selection based on only 269 SNPs (i.e. SNP_SSC7_) would result in greater response to selection for PRRS S/P, compared to using the whole genome (i.e. 38,191 SNPs in this study).

However, it should be noted that there is general concern about losing genetic variability in the MHC region [32]. Due to its major role in pathogen recognition, it is generally accepted that greater heterozygosity in the MHC region leads to detection and presentation of a wider range of antigens [33]. This suggests that individuals with greater MHC diversity have more chance to survive disease outbreaks but conflicting results in the literature indicate that this may not be a rule [32, 34, 35].

## Conclusions

Results from this study using field data show that PRRSV antibody response, measured as sample-to-positive ratio, had moderate to high heritabilities, with estimates increasing as the proportion of PRRSV-seropositive animals increased in the dataset. The two major QTL for S/P that were previously found on SSC7 (QTL_MHC_ and QTL_130_) were validated as being associated with S/P in all datasets. In addition, results from this study indicate that the magnitude of the PRRSV IgG response by S/P ratio can be predicted across populations and from S/P measured during acclimations and PPRSV vaccination to S/P measured in a natural reproductive PRRS outbreak with low to moderate accuracies using SNP genotypes. Moderate genomic prediction accuracies were obtained by using only the SNPs within the two major QTL regions, with overall accuracies across all scenarios of 0.39 and 0.31 for the outbreak and gilt acclimation datasets, respectively. SNPs in the QTL_MHC_ region showed overall greater predictive ability (0.34 for the outbreak and 0.25 for the gilt acclimation datasets) than SNPs in the QTL_130_ region (0.21 for the outbreak and 0.22 for the gilt acclimation datasets). In addition, genomic prediction accuracies using the rest of the genome (SNP_Rest_) indicated that SNPs not located within the two major QTL had little to no predictive ability for S/P. Overall, variable selection methods (Bayes-B and Bayes-C) resulted in greater genomic prediction accuracies than Bayesian GBLUP.

Our findings suggest that PRRSV antibody response (S/P ratio) in replacement gilts following standard acclimation procedures or in reproductive sows at approximately 45 days post-natural PRRSV infection can be predicted with high accuracy by using the SNPs that are included in just two QTL. Combined with our previous result, i.e. that S/P ratio is genetically correlated with reproductive performance during PRRS outbreak, this suggests that reproductive performance during PRRS infection can be selected for using PRRS S/P ratio or genomic predictions for S/P ratio, although further research is needed to validate these results. Due to the nature of the data, we were not able to assess the impact of using crossbred versus purebred animals for training or validation, or the impact of vaccination, or of the environment (production versus multiplier), or of the age of animals (gilts versus sows). Therefore, additional studies are needed to address these issues and better understand the role of these factors on the host genetics of antibody response to PRRSV.

## Additional files

10.1186/s12711-016-0230-0 Genomic prediction accuracies for different Bayesian methods, SNP sets, and sample-to-positive (S/P) ratio and validation datasets, including each fold of the cross-validation using the gilt acclimation (GA) dataset.
10.1186/s12711-016-0230-0Genomic prediction accuracies across genomic prediction methods, and SNP, and sample-to-positive (S/P) datasets for Fold 1, 2, 3, 4, 5, 6 and 7 (Figures S1, S2, S3, S4, S5, S6 and S7, respectively) of the seven-fold cross-validation using the gilt acclimation dataset. Results when using S/P datasets from S/P_0**%**_ to S/P_100**%**_ used for training are shown in panels designated by 0 % to 100 %, respectively. Within each column, color-coded bars represent genomic prediction accuracies for each method across SNP datasets. SNP datasets SNP_All_, SNP_MHC_, SNP_130_, SNP_SSC7_, and SNP_Rest_ are represented by All, MHC, 130, SSC7, and Rest, respectively.

## References

[1] Rowland RRR, Lunney JK, Dekkers JCM (2012). Control of porcine reproductive and respiratory syndrome (PRRS) through genetic improvements in disease resistance and tolerance. Front Genet.

[2] Lewis CR, Torremorell M, Galina-Pantoja L, Bishop SC (2009). Genetic parameters for performance traits in commercial sows estimated before and after an outbreak of porcine reproductive and respiratory syndrome. J Anim Sci.

[3] Serão NVL, Matika O, Kemp RA, Harding JC, Bishop SC, Plastow GS (2014). Genetic analysis of reproductive traits and antibody response in a PRRS outbreak herd. J Anim Sci.

[4] Gabler NK. The longitudinal impact of PRRS on metabolism, whole body protein accretion and feed efficiency in grow-finisher pigs. In: Proceedings of the ADSA-ASAS Midwest Meeting, 20–24 July 2014; Kansas City; 2014.

[5] Holtkamp DJK, Kliebenstein JB, Neumann EJ, Zimmerman JJ, Rotto HF, Yoder TK (2013). Assessment of the economic impact of porcine reproductive and respiratory syndrome virus on United States pork producers. J Swine Health Prod.

[6] Bottoms K, Poljak Z, Dewey C, Deardon R, Holtkamp D, Friendship R (2012). Investigation of strategies for the introduction and transportation of replacement gilts on southern Ontario sow farms. BMC Vet Res.

[7] Lambert M-È, Denicourt M, Poljak Z, D’Allaire S (2012). Gilt replacement strategies used in two swine production areas in Quebec in regard to porcine reproductive and respiratory syndrome virus. J Swine Health Prod.

[8] Orrett CM, Matika O, Archibald A, Lewis CRG, McLaren D, Deeb N (2013). Genetic of host response to infection with porcine reproductive and respiratory syndrome virus (PRRSv). Adv Anim Sci..

[9] Boddicker N, Waide EH, Rowland RR, Lunney JK, Garrick DJ, Reecy JM, Dekkers JC (2012). Evidence for a major QTL associated with host response to porcine reproductive and respiratory syndrome virus challenge. J Anim Sci.

[10] Serão NVL, Kemp RA, Mote BE, Harding JCS, Willson P, Bishop SC, et al. Whole-genome scan and validation of regions previously associated with PRRS antibody response and growth rate using gilts under health challenge in commercial settings. In: Proceedings of the 10th world congress of genetics applied to livestock production, 17–22 August 2014, Vancouver; 2014. https://asas.org/docs/default-source/wcgalp-proceedings-oral/103_paper_9221_manuscript_812_0.pdf?sfvrsn=2.

[11] Boddicker NJ, Bjorkquist A, Rowland RR, Lunney JK, Reecy JM, Dekkers JCM (2014). Genome-wide association and genomic prediction for host response to porcine reproductive and respiratory syndrome virus infection. Genet Sel Evol.

[12] Waide EH, Serão NVL, Hess AS, Rowland RRR, Lunney JK, PLastow GS, et al. Genome-wide association and genomic prediction of response to infection for two isolates of porcine reproductive and respiratory syndrome virus. In: Proceedings of the ADSA-ASAS midwest meeting, 16–18 March 2015, Des Moines; 2015.

[13] Canadian Council on Animal Care. Guide to the care and use of experimental animals. Vol. 1, 3rd ed. Ottawa: Canadian Council on Animal Care; 1993.

[14] VanRaden PM (2008). Efficient methods to compute genomic predictions. J Dairy Sci.

[15] Gilmour AR, Gogel BJ, Cullis BR, Welham SJ, Thompson R. ASReml user guide release 4.1. 2015. http://www.vsni.co.uk/downloads/asreml/release4/UserGuideStructural.pdf. Accessed 10 Feb 2016.

[16] Habier D, Fernando RL, Kizilkaya K, Garrick DJ (2011). Extension of the Bayesian alphabet for genomic selection. BMC Bioinformat.

[17] Fernando RL, Garrick DJ. GenSel manual v3. 2009. http://www.biomedcentral.com/content/supplementary/1471-2105-12-186-s1.pdf. 2009. Accessed 10 Feb 2016.

[18] Garrick DJ, Fernando RL (2013). Implementing a QTL detection study (GWAS) using genomic prediction methodology. Methods Mol Biol.

[19] Saitou N, Nei M (1987). The neighbor-joining method: a new method for reconstructing phylogenetic trees. Mol Biol Evol.

[20] Nei M (1972). Genetic distance between populations. Am Nat.

[21] Pembleton LW, Cogan NO, Forster JW (2013). StAMPP: an R package for calculation of genetic differentiation and structure of mixed-ploidy level populations. Mol Ecol Resour.

[22] Tamura K, Stecher G, Peterson D, Filipski A, Kumar S (2013). MEGA6: molecular evolutionary genetics analysis version 6.0. Mol Biol Evol.

[23] Lunney JK, Ho CS, Wysocki M, Smith DM (2009). Molecular genetics of the swine major histocompatibility complex, the SLA complex. Dev Comp Immunol.

[24] Engle TB, Jobman EE, Moural TW, McKnite AM, Bundy JW, Barnes SY (2014). Variation in time and magnitude of immune response and viremia in experimental challenges with Porcine circovirus 2b. BMC Vet Res.

[25] Dunkelberger JR, Waide EH, Serão NVL, Lunney JK, Rowland RRR, Dekkers JCM. Genomic prediction of host response to co-infection with PRRSV and PCV2b using a PRRSV-only infected training population. In: Proceedings of the ADSA-ASAS midwest meeting, 16–18 March 2015, Des Moines; 2015.

[26] Hess AS, Trible BR, Wang Y, Boddicker NJ, Rowland RRR, Lunney JK, et al. Identification of a major QTL associated with N-specific IgG response in piglets experimentally infected with porcine reproductive and respiratory syndrome virus. In: Proceedings of the ADSA-ASAS joint annual meeting, 8–12 July 2013, Indianapolis; 2013.

[27] Thompson-Crispi KA, Sargolzaei M, Ventura R, Abo-Ismail M, Miglior F, Schenkel F (2014). A genome-wide association study of immune response traits in Canadian Holstein cattle. BMC Genomics.

[28] Lin WW, Hildebrand JM, Bishop GA (2013). A complex relationship between TRAF3 and non-canonical NF-kappaB2 activation in B lymphocytes. Front Immunol.

[29] Ellingson JS, Wang Y, Layton S, Ciacci-Zanella J, Roof MB, Faaberg KS (2010). Vaccine efficacy of porcine reproductive and respiratory syndrome virus chimeras. Vaccine.

[30] Bishop SC, Woolliams JA (2010). On the genetic interpretation of disease data. PLoS One.

[31] Liu T, Qu H, Luo C, Li X, Shu D, Lund MS (2014). Genomic selection for the improvement of antibody response to Newcastle disease and avian influenza virus in chickens. PLoS One.

[32] Ellis SA, Hammond JA (2014). The functional significance of cattle major histocompatibility complex class I genetic diversity. Annu Rev Anim Biosci.

[33] Sommer S (2005). The importance of immune gene variability (MHC) in evolutionary ecology and conservation. Front Zool.

[34] Parham P, Ohta T (1996). Population biology of antigen presentation by MHC class I molecules. Science.

[35] Gangoso L, Alcaide M, Grande JM, Munoz J, Talbot SL, Sonsthagen SA (2012). Colonizing the world in spite of reduced MHC variation. J Evol Biol.

