# Supplementary material for: Genetic and genomic basis of antibody response to porcine reproductive and respiratory syndrome (PRRS) in gilts and sows
Source: Genet Sel Evol. 2016 Jul 14;48:51. doi: 10.1186/s12711-016-0230-0 (PMC4944421; doi:10.1186/s12711-016-0230-0)
Supplement: Supplementary file 1 — 10.1186/s12711-016-0230-0 Genomic prediction accuracies for different Bayesian methods, SNP sets, and sample-to-positive (S/P) ratio and validation datasets, including each fold of the cross-validation using the gilt acclimation (GA) dataset. [file 12711_2016_230_MOESM1_ESM.docx]

|  | SNP dataset | | | | | | | | | | | | | | | | | | | |
| --- | --- | --- | --- | --- | --- | --- | --- | --- | --- | --- | --- | --- | --- | --- | --- | --- | --- | --- | --- | --- |
|  |  | All | | |  | MHC | | |  | 130 | | |  | SSC7 | | |  | Rest | | |
| Validation | S/P_(%)_^1^ | Bayes-B | Bayes-C | GBLUP |  | Bayes-B | Bayes-C | GBLUP |  | Bayes-B | Bayes-C | GBLUP |  | Bayes-B | Bayes-C | GBLUP |  | Bayes-B | Bayes-C | GBLUP |
| Outbreak | 0 | 0.45 | 0.41 | 0.08 |  | 0.40 | 0.40 | 0.30 |  | 0.26 | 0.26 | 0.14 |  | 0.48 | 0.48 | 0.31 |  | 0.10 | 0.09 | 0.02 |
|  | 25 | 0.44 | 0.41 | 0.08 |  | 0.40 | 0.40 | 0.30 |  | 0.26 | 0.26 | 0.11 |  | 0.48 | 0.48 | 0.31 |  | 0.10 | 0.09 | 0.02 |
|  | 50 | 0.34 | 0.30 | 0.06 |  | 0.39 | 0.38 | 0.26 |  | 0.27 | 0.27 | 0.26 |  | 0.48 | 0.46 | 0.29 |  | 0.01 | 0.01 | 0.00 |
|  | 75 | 0.35 | 0.33 | 0.09 |  | 0.38 | 0.37 | 0.25 |  | 0.25 | 0.25 | 0.05 |  | 0.46 | 0.45 | 0.25 |  | 0.08 | 0.08 | 0.04 |
|  | 100 | 0.33 | 0.29 | 0.02 |  | 0.31 | 0.32 | 0.21 |  | 0.24 | 0.24 | -0.02 |  | 0.38 | 0.39 | 0.21 |  | 0.11 | 0.09 | -0.03 |
| GA | 0 | 0.33 | 0.33 | 0.15 |  | 0.24 | 0.26 | 0.28 |  | 0.26 | 0.26 | 0.14 |  | 0.33 | 0.35 | 0.29 |  | 0.09 | 0.10 | 0.09 |
|  | 25 | 0.33 | 0.33 | 0.16 |  | 0.25 | 0.26 | 0.28 |  | 0.26 | 0.26 | 0.14 |  | 0.33 | 0.35 | 0.30 |  | 0.09 | 0.10 | 0.10 |
|  | 50 | 0.35 | 0.36 | 0.18 |  | 0.25 | 0.27 | 0.27 |  | 0.25 | 0.25 | 0.15 |  | 0.33 | 0.35 | 0.29 |  | 0.14 | 0.14 | 0.11 |
|  | 75 | 0.38 | 0.38 | 0.20 |  | 0.25 | 0.27 | 0.27 |  | 0.26 | 0.26 | 0.13 |  | 0.34 | 0.36 | 0.29 |  | 0.18 | 0.18 | 0.13 |
|  | 100 | 0.27 | 0.29 | 0.18 |  | 0.15 | 0.18 | 0.24 |  | 0.24 | 0.25 | 0.11 |  | 0.23 | 0.26 | 0.25 |  | 0.16 | 0.17 | 0.13 |
| Fold 1 | 0 | 0.21 | 0.25 | 0.27 |  | 0.19 | 0.22 | 0.22 |  | -0.03 | -0.03 | -0.04 |  | 0.11 | 0.14 | 0.21 |  | 0.20 | 0.23 | 0.23 |
|  | 25 | 0.22 | 0.27 | 0.27 |  | 0.18 | 0.21 | 0.22 |  | -0.03 | -0.03 | -0.03 |  | 0.11 | 0.14 | 0.21 |  | 0.23 | 0.27 | 0.23 |
|  | 50 | 0.24 | 0.28 | 0.30 |  | 0.18 | 0.20 | 0.21 |  | -0.03 | -0.03 | -0.03 |  | 0.13 | 0.14 | 0.20 |  | 0.23 | 0.26 | 0.26 |
|  | 75 | 0.27 | 0.29 | 0.30 |  | 0.16 | 0.18 | 0.20 |  | -0.01 | -0.01 | 0.07 |  | 0.11 | 0.14 | 0.21 |  | 0.28 | 0.28 | 0.26 |
|  | 100 | 0.22 | 0.26 | 0.20 |  | 0.16 | 0.17 | 0.19 |  | -0.02 | -0.02 | 0.06 |  | 0.10 | 0.13 | 0.20 |  | 0.25 | 0.25 | 0.15 |
| Fold 2 | 0 | 0.53 | 0.54 | 0.19 |  | 0.25 | 0.29 | 0.38 |  | 0.52 | 0.52 | 0.27 |  | 0.47 | 0.52 | 0.43 |  | 0.23 | 0.23 | 0.10 |
|  | 25 | 0.51 | 0.53 | 0.23 |  | 0.27 | 0.33 | 0.38 |  | 0.51 | 0.50 | 0.28 |  | 0.47 | 0.52 | 0.43 |  | 0.21 | 0.20 | 0.14 |
|  | 50 | 0.52 | 0.54 | 0.23 |  | 0.26 | 0.29 | 0.34 |  | 0.43 | 0.45 | 0.35 |  | 0.41 | 0.45 | 0.39 |  | 0.30 | 0.29 | 0.14 |
|  | 75 | 0.53 | 0.54 | 0.21 |  | 0.25 | 0.31 | 0.36 |  | 0.49 | 0.48 | 0.31 |  | 0.45 | 0.50 | 0.42 |  | 0.29 | 0.26 | 0.12 |
|  | 100 | 0.40 | 0.39 | 0.19 |  | 0.20 | 0.20 | 0.29 |  | 0.44 | 0.44 | 0.20 |  | 0.35 | 0.36 | 0.34 |  | 0.22 | 0.20 | 0.11 |
| Fold 3 | 0 | 0.17 | 0.14 | -0.20 |  | 0.14 | 0.16 | 0.12 |  | 0.42 | 0.43 | 0.57 |  | 0.34 | 0.36 | 0.23 |  | -0.17 | -0.19 | -0.25 |
|  | 25 | 0.17 | 0.15 | -0.18 |  | 0.16 | 0.18 | 0.13 |  | 0.42 | 0.43 | 0.56 |  | 0.33 | 0.36 | 0.23 |  | -0.16 | -0.17 | -0.24 |
|  | 50 | 0.23 | 0.24 | -0.16 |  | 0.17 | 0.22 | 0.13 |  | 0.43 | 0.43 | 0.53 |  | 0.35 | 0.40 | 0.20 |  | -0.04 | -0.04 | -0.21 |
|  | 75 | 0.26 | 0.24 | -0.21 |  | 0.22 | 0.26 | 0.15 |  | 0.43 | 0.44 | 0.46 |  | 0.42 | 0.46 | 0.22 |  | -0.08 | -0.12 | -0.26 |
|  | 100 | 0.21 | 0.14 | -0.23 |  | 0.31 | 0.32 | 0.14 |  | 0.45 | 0.45 | 0.47 |  | 0.49 | 0.50 | 0.22 |  | -0.20 | -0.21 | -0.28 |
| Fold 4 | 0 | 0.27 | 0.30 | 0.17 |  | 0.20 | 0.27 | 0.21 |  | 0.19 | 0.20 | 0.32 |  | 0.26 | 0.32 | 0.26 |  | 0.10 | 0.10 | 0.11 |
|  | 25 | 0.28 | 0.29 | 0.16 |  | 0.20 | 0.24 | 0.21 |  | 0.19 | 0.20 | 0.31 |  | 0.26 | 0.30 | 0.25 |  | 0.12 | 0.12 | 0.10 |
|  | 50 | 0.30 | 0.32 | 0.15 |  | 0.21 | 0.26 | 0.22 |  | 0.19 | 0.19 | 0.34 |  | 0.26 | 0.31 | 0.26 |  | 0.14 | 0.13 | 0.09 |
|  | 75 | 0.33 | 0.35 | 0.19 |  | 0.19 | 0.25 | 0.23 |  | 0.23 | 0.23 | 0.26 |  | 0.27 | 0.32 | 0.27 |  | 0.19 | 0.18 | 0.13 |
|  | 100 | 0.07 | 0.17 | 0.18 |  | -0.11 | -0.03 | 0.20 |  | 0.21 | 0.22 | 0.25 |  | 0.00 | 0.08 | 0.24 |  | 0.13 | 0.17 | 0.12 |
| Fold 5 | 0 | 0.32 | 0.30 | 0.14 |  | 0.17 | 0.17 | 0.31 |  | 0.39 | 0.38 | -0.08 |  | 0.35 | 0.34 | 0.29 |  | 0.05 | 0.06 | 0.07 |
|  | 25 | 0.33 | 0.30 | 0.14 |  | 0.21 | 0.20 | 0.31 |  | 0.39 | 0.38 | -0.07 |  | 0.37 | 0.36 | 0.29 |  | 0.04 | 0.05 | 0.07 |
|  | 50 | 0.36 | 0.33 | 0.18 |  | 0.25 | 0.25 | 0.31 |  | 0.39 | 0.39 | -0.02 |  | 0.39 | 0.39 | 0.31 |  | 0.08 | 0.07 | 0.09 |
|  | 75 | 0.39 | 0.36 | 0.25 |  | 0.29 | 0.27 | 0.32 |  | 0.37 | 0.36 | -0.10 |  | 0.43 | 0.40 | 0.29 |  | 0.12 | 0.13 | 0.18 |
|  | 100 | 0.32 | 0.32 | 0.33 |  | 0.14 | 0.15 | 0.29 |  | 0.39 | 0.38 | -0.05 |  | 0.26 | 0.28 | 0.27 |  | 0.20 | 0.20 | 0.27 |
| Fold 6 | 0 | 0.40 | 0.37 | 0.17 |  | 0.38 | 0.38 | 0.38 |  | 0.25 | 0.25 | 0.08 |  | 0.44 | 0.44 | 0.39 |  | 0.04 | 0.05 | 0.09 |
|  | 25 | 0.38 | 0.36 | 0.17 |  | 0.38 | 0.38 | 0.38 |  | 0.25 | 0.25 | 0.09 |  | 0.44 | 0.44 | 0.39 |  | 0.02 | 0.03 | 0.10 |
|  | 50 | 0.42 | 0.40 | 0.20 |  | 0.39 | 0.39 | 0.37 |  | 0.23 | 0.23 | 0.05 |  | 0.44 | 0.43 | 0.37 |  | 0.08 | 0.09 | 0.11 |
|  | 75 | 0.47 | 0.46 | 0.21 |  | 0.37 | 0.38 | 0.36 |  | 0.25 | 0.25 | 0.07 |  | 0.43 | 0.44 | 0.37 |  | 0.19 | 0.19 | 0.13 |
|  | 100 | 0.39 | 0.38 | 0.21 |  | 0.30 | 0.31 | 0.31 |  | 0.18 | 0.19 | -0.02 |  | 0.34 | 0.35 | 0.30 |  | 0.20 | 0.19 | 0.15 |
| Fold 7 | 0 | 0.82 | 0.82 | 0.63 |  | 0.14 | 0.05 | -0.38 |  | -0.20 | -0.20 | -0.08 |  | 0.04 | -0.06 | -0.43 |  | 0.89 | 0.86 | 0.71 |
|  | 25 | 0.81 | 0.79 | 0.63 |  | 0.13 | 0.05 | -0.37 |  | -0.19 | -0.19 | -0.08 |  | 0.04 | -0.06 | -0.42 |  | 0.89 | 0.85 | 0.71 |
|  | 50 | 0.45 | 0.44 | 0.52 |  | -0.09 | -0.12 | -0.38 |  | -0.19 | -0.19 | -0.10 |  | -0.21 | -0.24 | -0.43 |  | 0.59 | 0.57 | 0.63 |
|  | 75 | 0.19 | 0.23 | 0.49 |  | -0.24 | -0.22 | -0.41 |  | -0.23 | -0.23 | -0.11 |  | -0.39 | -0.38 | -0.47 |  | 0.49 | 0.48 | 0.61 |
|  | 100 | 0.19 | 0.29 | 0.12 |  | -0.17 | -0.15 | -0.37 |  | -0.23 | -0.23 | -0.25 |  | -0.37 | -0.34 | -0.45 |  | 0.45 | 0.48 | 0.27 |

^1^Numbers from 0 to 100 represent the minimum percentage of PRRSV-seropositive animals within contemporary group used for each S/P dataset.
