# Supplementary figures and images for: Genetic and genomic basis of antibody response to porcine reproductive and respiratory syndrome (PRRS) in gilts and sows
Source: Genet Sel Evol. 2016 Jul 14;48:51. doi: 10.1186/s12711-016-0230-0 (PMC4944421; doi:10.1186/s12711-016-0230-0)

Figure S1

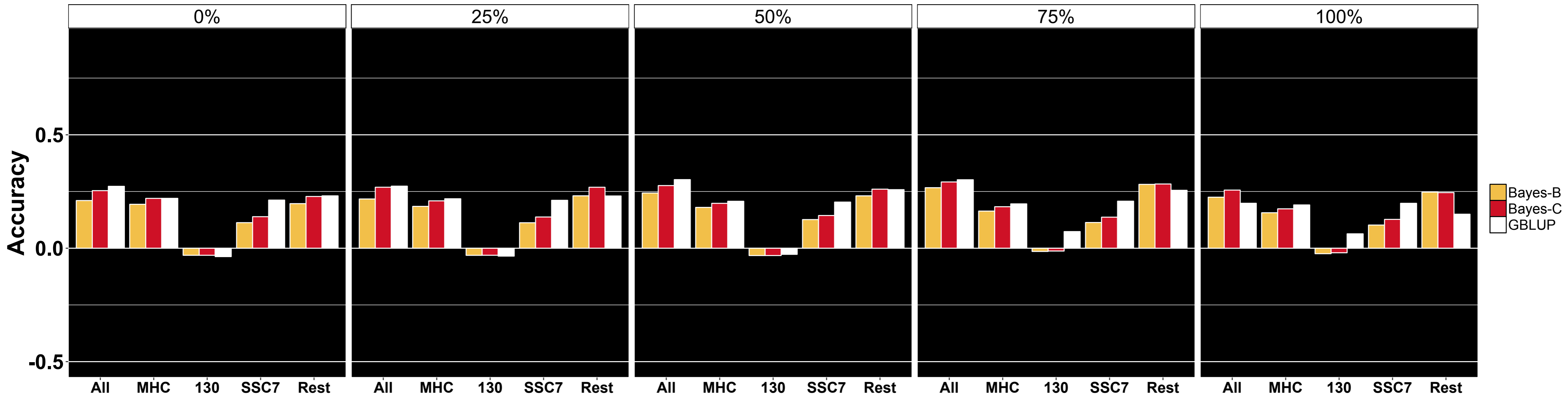

Figure S2

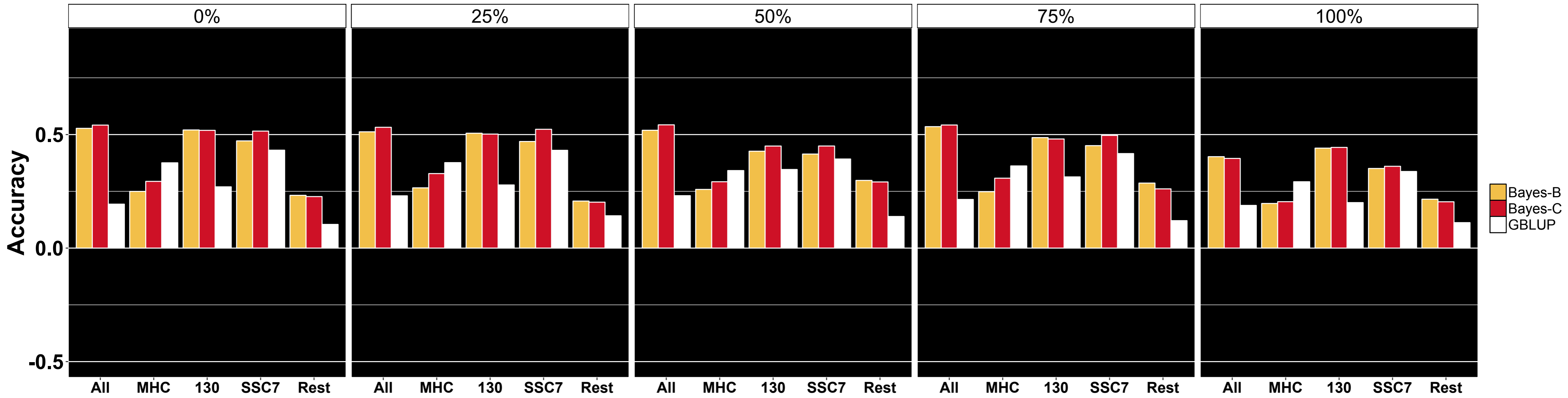

Figure S3

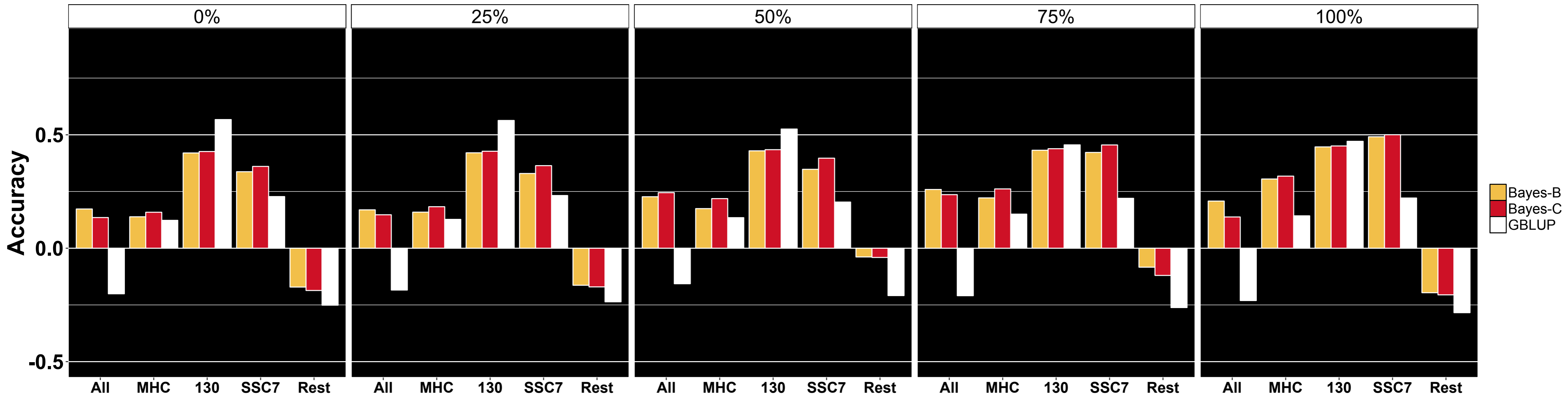

Figure S4

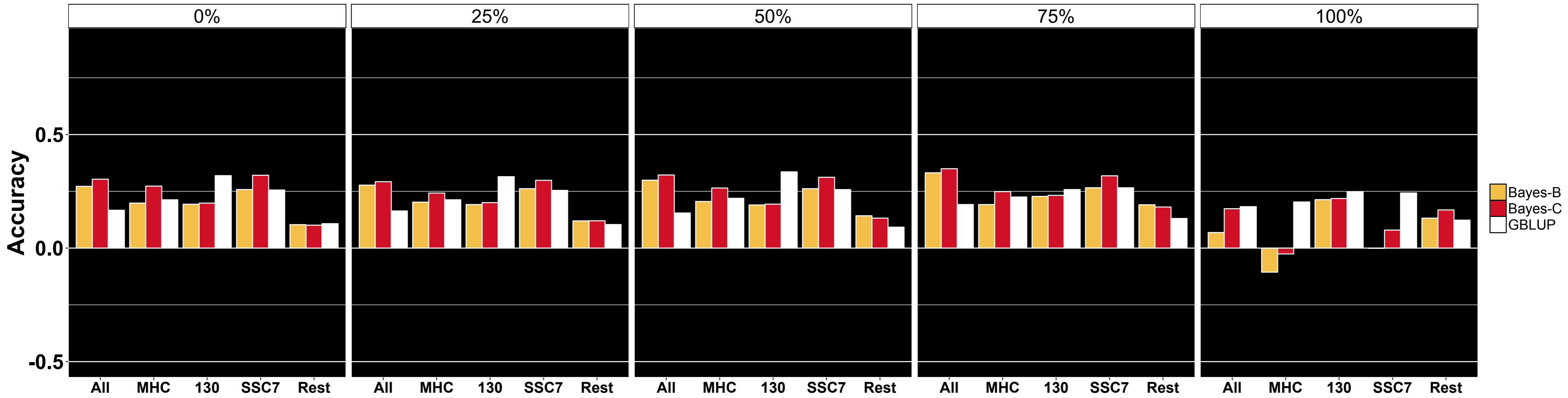

Figure S5

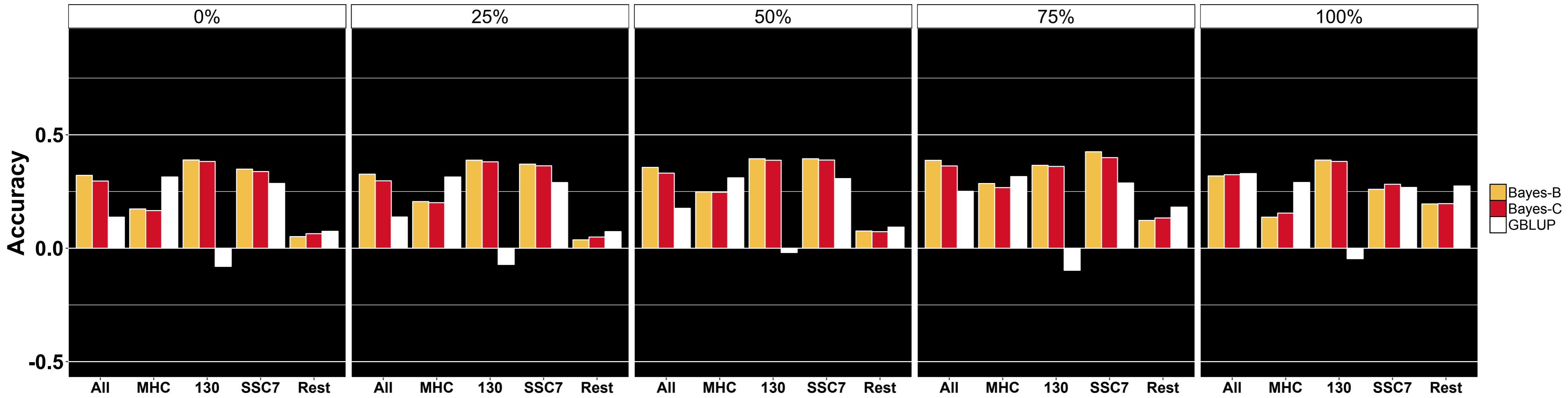

Figure S6

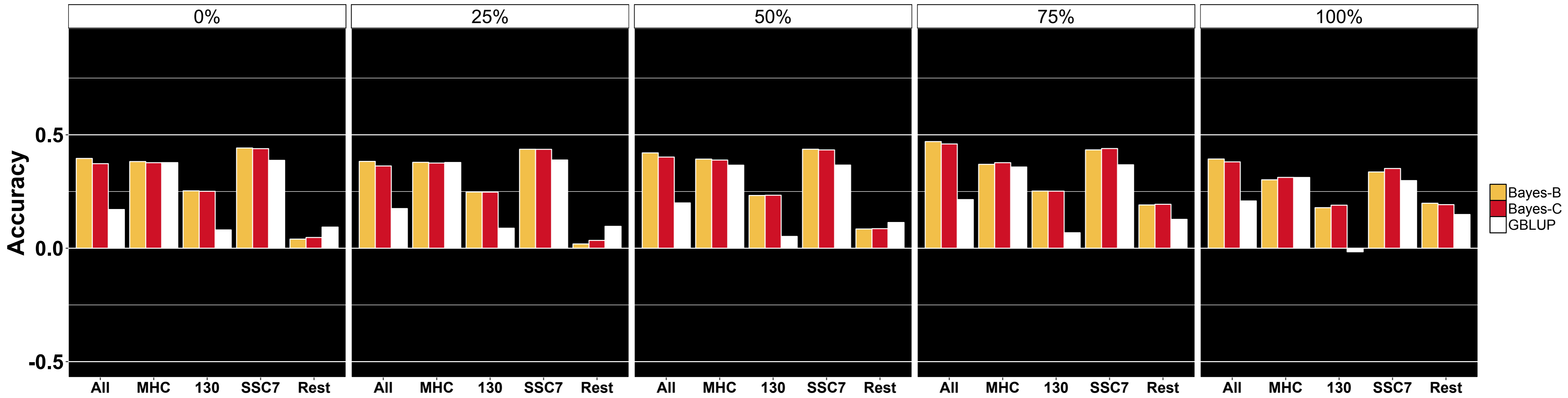

Figure S7

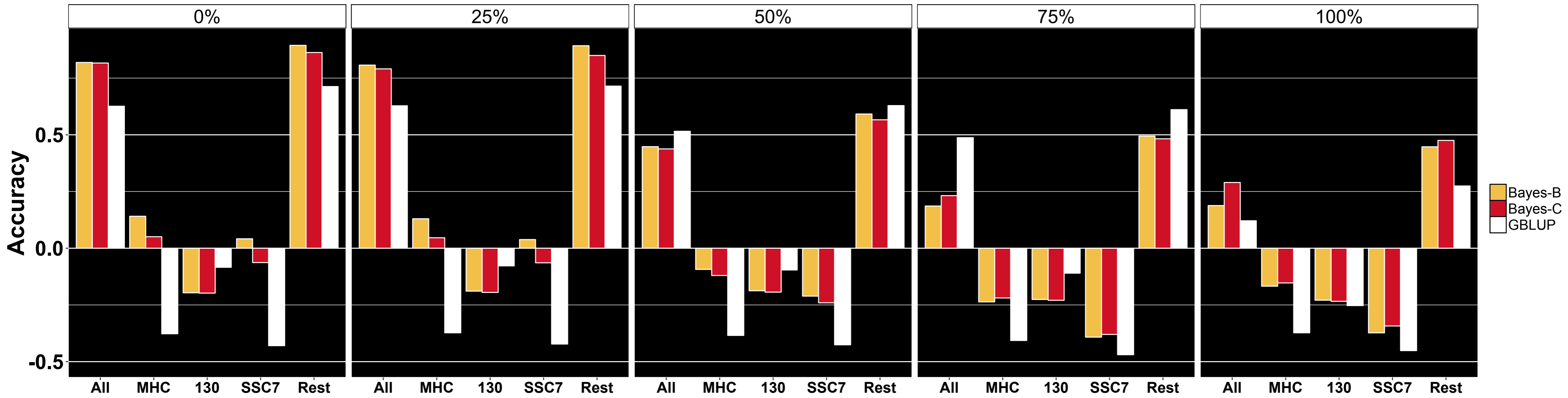

Supplement: Supplementary file 2 — 10.1186/s12711-016-0230-0Genomic prediction accuracies across genomic prediction methods, and SNP, and sample-to-positive (S/P) datasets for Fold 1, 2, 3, 4, 5, 6 and 7 (Figures S1, S2, S3, S4, S5, S6 and S7, respectively) of the seven-fold cross-validation using the gilt acclimation dataset. Results when using S/P datasets from S/P0% to S/P100% used for training are shown in panels designated by 0 % to 100 %, respectively. Within each column, color-coded bars represent genomic prediction accuracies for each method across SNP datasets. SNP datasets SNPAll, SNPMHC, SNP130, SNPSSC7, and SNPRest are represented by All, MHC, 130, SSC7, and Rest, respectively. [file 12711_2016_230_MOESM2_ESM.pdf]
